# Chronic sleep deprivation altered the expression of circadian clock genes and aggravated Alzheimer's disease neuropathology

**DOI:** 10.1111/bpa.13028

**Published:** 2021-10-20

**Authors:** Long Niu, Feng Zhang, Xiaojiao Xu, Yuting Yang, Song Li, Hui Liu, Weidong Le

**Affiliations:** ^1^ Center for Clinical Research on Neurological Diseases the First Affiliated Hospital Dalian Medical University Dalian China; ^2^ Liaoning Provincial Key Laboratory for Research on the Pathogenic Mechanisms of Neurological Diseases the First Affiliated Hospital Dalian Medical University Dalian China; ^3^ Department of Neurology Minhang Hospital Fudan University Shanghai China; ^4^ Department of Neurology and Institute of Neurology Sichuan Academy of Medical Science‐Sichuan Provincial Hospital Chengdu China

**Keywords:** Alzheimer's disease, circadian rhythm, clock genes, sleep disorders

## Abstract

Circadian disruption is prevalent in Alzheimer's disease (AD) and may contribute to cognitive impairment, psychological symptoms, and neurodegeneration. This study aimed to evaluate the effects of environmental and genetic factors on the molecular clock and to establish a link between circadian rhythm disturbance and AD. We investigated the pathological effects of chronic sleep deprivation (CSD) in the APP^swe^/PS1^ΔE9^ transgenic mice and their wild‐type (WT) littermates for 2 months and evaluated the expression levels of clock genes in the circadian rhythm‐related nuclei. Our results showed that CSD impaired learning and memory, and further exaggerated disease progression in the AD mice. Furthermore, CSD caused abnormal expression of Bmal1, Clock, and Cry1 in the circadian rhythm‐related nuclei of experimental mice, and these changes are more significant in AD mice. Abnormal expression of clock genes in AD mice suggested that the expression of clock genes is affected by APP/PS1 mutations. In addition, abnormal tau phosphorylation was found in the retrosplenial cortex, which was co‐located with the alteration of BMAL1 protein level. Moreover, the level of tyrosine hydroxylase in the locus coeruleus of AD and WT mice was significantly increased after CSD. There may be a potential link between the molecular clock, Aβ pathology, tauopathy, and the noradrenergic system. The results of this study provided new insights into the potential link between the disruption of circadian rhythm and the development of AD.

## Graphical abstract

CSD exaggerated disease progression in the AD mice. After CSD, the abnormal expression of clock genes in AD mice pineal gland, LC and RSC may be related to the dysfunction of SCN. The level of tyrosine hydroxylase in the locus coeruleus of AD and WT mice was significantly increased after CSD. There may be a potential link between the molecular clock, Aβ pathology, tauopathy, and noradrenergic system.

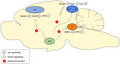

**Abbreviations**
AD: Alzheimer's disease
Aβ: amyloid‐β
BMAL1: brain and muscle arnt‐like protein‐1
CLOCK: circadian locomotor output cycles kaput
CRY: cryptochrome circadian regulator
CSD: chronic sleep deprivation
LC: locus coeruleus
MMP: modified multiple platform
NE: norepinephrine
NORT: novel object recognition task
PBS: phosphate‐buffer saline
PER: period circadian regulator
p‐Tau: phospho‐Tau
REM: rapid eye movement
RSC: retrosplenial cortex
SCN: suprachiasmatic nucleus
TH: tyrosine hydroxylase

## 1 INTRODUCTION

Alzheimer's disease (AD) is the most common form of dementia among the older population. It is characterized by amyloid‐β (Aβ) deposition, abnormal tau phosphorylation, and neuronal loss in the brain. Aging is associated with many health problems, including difficult sleep. Among the elderly, 23% to 34% had symptoms of insomnia [1]. Another study reported that subjective sleep complaints predict cognitive decline in middle aged and older adults [2]. Sleep disorders in elderly people may be an early sign of age‐related neurodegenerative diseases and mild cognitive impairment. As reported, 24.5%–40% of patients with AD have chronic sleep disorders [3]. Another study indicated that over 60% of the patients with mild cognitive impairment and AD suffer from at least one type of sleep disturbance [4]. Sleep disorders appear early in patients with AD and worsen as the disease progresses, which is considered a strong predictor of mortality in the late stages of AD [5]. Memory decline in AD could be a consequence of impairment in sleep‐dependent memory consolidation [6]. It is proposed that the recently acquired memories in the hippocampal network are reprocessed offline during sleep [7]. A study has reported that sleep‐dependent memory consolidation is impaired in both aging and AD [8]. Therefore, sleep disorders may be a potential cause of memory loss, not just a consequence of the pathological progression of AD.

Sleep disorders in AD are thought to be caused by the altered regulation of circadian rhythm, changes in neuroendocrine mediators, and impairment of the interstitial fluid clearance system in the brain [9]. In the late stage of AD, the expression of MT1 melatonin receptor in the suprachiasmatic nucleus (SCN) is significantly reduced [10]. Studies on animal models have also revealed the possible pathological association between AD and altered circadian rhythms. Circadian rhythm changes in 3xTG‐AD mice usually present before the expected AD pathology [11]. It has been reported that activity changes and marked stereotypic behavior preceded Aβ pathology in TgCRND8 mice [12]. A study on the circadian rhythm in APP/PS1 mice showed that the most significant effects of the APPxPS1 transgenes were phase delays of ~2 h in the onset of daytime wakefulness bouts and peak wakefulness, which potentially relevant to phase delays previously reported in AD patients [13]. These studies suggested that the circadian rhythm disruption may occur before the pathological changes of AD and affect disease development.

The circadian rhythm is regulated by a series of circadian rhythm‐related nuclei and hormones. The SCN is the master circadian pacemaker, and the overall SCN volume has been reported to be reduced in patients with AD [14]. The SCN receives the information about daily light exposure from the retina and then communicates the time information to other peripheral oscillators in various brain regions and organs through synaptic and diffusible signals [15]. The locus coeruleus (LC) is the primary source of norepinephrine (NE) for the central nervous system. LC noradrenergic neurons innervate the spinal cord, brainstem, thalamus, cerebellum, and most of the cortex [16, 17, 18]. The alteration to LC and its projections may disrupt cognitive functions, including arousal, attention, learning, and memory [19]. Studies have suggested that the neurofibrillary tangle formation in the LC is an early event of AD [20, 21]. The retrosplenial cortex (RSC) has emerged as a key brain area that supports memory, including episodic and topographical memory in humans and spatial memory in rodents [19]. RSC is consistently compromised in most common neurological disorders with impaired memory [22]. The earliest metabolic decline in AD and mild cognitive impairment is centered on the RSC, which is consistent with the relationship between retrosplenial hypoactivity and memory loss in patients with early AD [23, 24, 25]. Moreover, RSC also regulates the cortical activation during rapid eye movement (REM) sleep, and its dysfunction may lead to REM sleep behavior disorder [26].

The circadian rhythm is regulated by a group of genes, including the positive regulators brain and muscle arnt‐like protein‐1 (BMAL1), circadian locomotor output cycles kaput (CLOCK), cryptochrome circadian regulator (CRY)1/2, and period circadian regulator (PER)1/2/3 [27]. BMAL1 heterodimerizes with CLOCK binds to the E‐box motif in the entire genome and drives transcription of a group of genes [28]. PER and CRY proteins enter the nucleus and inhibit CLOCK and BMAL1 to regulate their own transcription [29], forming the core negative limb of the transcriptional/translational feedback loop. Bmal1, Cry1, and Per1 were reported to have rhythmic expression in the human pineal gland, and these rhythms are lost in patients with preclinical and clinical AD [10]. As reported, Aβ dynamics were regulated by orexin and the sleep‐wake cycle [30]. The circadian rhythm and its related regulatory nuclei and genes are possibly linked to AD. Our group previously reported that chronic sleep deprivation (CSD) can cause learning and memory impairment and aggravate the pathological changes in AD [31]. The modified multiple platform (MMP) method was used to interrupt the sleep rhythms, especially the REM sleep, which caused SD [32]. To investigate the effect of CSD on the molecular clock and its possible association with AD pathology, we established a CSD mouse model by MMP method, and then determined the expression of clock genes in mouse RSC, LC, and pineal gland after CSD, and analyzed the potential relationship between the expression of clock genes and pathological changes of AD.

## 2 METHODS

### 2.1 Experimental animals and chronic sleep deprivation

APP^swe^/PS1^ΔE9^ AD mice for breeding were purchased from the Jackson Laboratory (B6C3‐Tg (AβPPswe, PSEN1dE9)85Dbo/Mmjax, Bar Harbor, MA, USA). AD and WT mice were kept under 12–12 h light–dark cycle (lights on at 7:00 AM), room temperature of 22°C ± 1°C, and relative humidity of 50% ± 10%. The APP/PS1 mice develop Aβ deposits in brain by 6–7 months of age. AD and WT male mice aged 4–4.5 months were randomly divided into four groups (*n* = 10 in each group): AD with CSD (AD + CSD), AD with non‐CSD (AD + NSD), WT with CSD (WT + CSD), and WT with non‐CSD (WT + NSD). AD + CSD and WT + CSD mice were subjected to CSD for 2 months using the MMP method (from 14:00 to 10:00 the next day) [31] (Figure S1). During the CSD process, the mice were placed in a water tank filled with 22°C water. There were 24 small platforms with a diameter of 3 cm and a height of 5 cm, on which the mice could only stand because of the instinctive fear of water; once asleep, the mice would fall into the water. AD + NSD and WT + NSD mice were placed in a water tank with a platform diameter of 11.5 cm; other conditions were similar to those of CSD groups. Food and water were available ad libitum throughout the studies. After 2 months of CSD treatment, all the mice were 6–6.5 months old. All mice were subjected to behavioral tests to evaluate their learning and memory ability and then sacrificed for brain sampling and pathological assessments at 9:30 AM. Animal care and procedures were carried out in accordance with the Laboratory Animal Care Guidelines approved by the Institutional Animal Care Committee at Dalian Medical University. The protocol was approved by the Institutional Animal Care Committee at Dalian Medical University. Every effort was made to minimize animal suffering. The minimum number of animals necessary to obtain statistically reliable data was utilized.

### 2.2 Behavioral tests

#### 2.2.1 Morris water maze

Morris water maze was conducted to test the learning and memory of mice after CSD. Training was conducted for 4 days in a circular sink, which was divided into four quadrants. In one quadrant, there was a hidden platform (12 cm in diameter) that was beneath the surface of the water. The mice were randomly placed into four starting quadrants to swim freely on each training day. In each trial, mice swam until they found the hidden platform or were guided to it by the experimenter if not found within 90 s. When the mice arrived at the platform, they stayed on the platform for 30 s. A probe trial was conducted on day 5, the hidden platform was removed, and the mice swam from the farthest quadrant of the platform for a total of 90 s. The swimming track of the mice was recorded. The experiment was carried out using a recording camera and analysis system (SOF‐845, Med Associates, USA).

#### 2.2.2 Y maze

The Y maze was created in a Y‐shaped container with three arms, marked as A, B, and C arms. The mice were placed in the middle and allowed to explore in the Y maze for 6 min, and the order in which the mice entered each arm was recorded by the camera recording system and Supermaze software (XR‐Xmaze, Shanghai Xinruan Information Technology Co., Ltd, China). If the mice entered three different arms, it was considered as one “correct” recording, for example, A‐B‐C, A‐C‐B, B‐C‐A, B‐A‐C, C‐A‐B, and C‐B‐A. The Y‐maze alternation rate was calculated as follows: *R* (%) = *M*/(*N* − 2) × 100%, where M was the “correct” recording number, N was the total number of entering each arm, and *N* > 10 can be included in the data.

#### 2.2.3 Novel object recognition task

Novel object recognition task (NORT) was performed in a plastic, open field arena (50 × 35 × 20 cm^3^). On the first day, the mice were adapted to the cabin for 10 min, and on the second day, two same balls A and B (7.5 cm in diameter) were placed in the middle of the cabin (15 cm from the wall; the distance between the two objects was 20 cm). Then, the mice were allowed to explore ball A and ball B for 5 min. When the mouse nose was sniffing object <1 cm, it was considered as exploring the object. On the third day, ball B was replaced with conical C (bottom 7.5 cm), and the mice were placed in the cabin to explore the object for 5 min. After 1 h, the position was swapped between A and C, and the mice were placed into the cabin to explore the objects for 5 min. The total time spent exploring the objects during each phase was recorded. NORT discrimination ratio was calculated as follows: *R*% = *T*_new_/(*T*_new_ + *T*_familiar_) × 100%, where *T*_new_ was the time of investigating new objects (conical C), *T*_familiar_ was the time of investigating familiar objects (ball A), and the total time spent investigating the objects was limited to a minimum of 10 s.

### 2.3 Real‐time quantitative polymerase chain reaction

Protocols for total RNA extraction, cDNA synthesis, and quantitative polymerase chain reaction (qPCR) were described previously [33]. After CSD, experimental mice were anesthetized and perfused with cold 0.1 M phosphate‐buffer saline (PBS) and 4% paraformaldehyde through the vascular system, and their brains were removed subsequently. The tissues were collected from the pineal gland by manual microdissection (bregma −3.88 mm). Total RNA was extracted from the pineal gland (*n* = 3 in each group) by RNAiso Plus (Total RNA extraction reagent; Takara, Shiga, Japan). The total RNA was synthesized to cDNA according to the instructions of the Revertra ACE QPCR RT kit (Takara, Shiga, Japan). qPCR was performed using TransStart Top Green qPCR SuperMix (TransGen Biotech, Co., Ltd, Beijing, China) and monitored by the real‐time PCR system (Applied Biosystems 7500 Real‐Time PCR Systems). The sequence of primers is described in Table 1. The relative expression level of each primer sequence was analyzed by 2^−ΔΔCt^ algorithm normalized to glyceraldehyde 3‐phosphate dehydrogenase and compared with the control groups.

**TABLE 1:** Primers sequences for real‐time PCR.

	Forward (5′‐3′)	Reverse (5′‐3′)	Gene access number
*Gapdh*	TGTGTCCGTCGTGGATCTGA	TTGCTGTTGAAGTCGCAGGAG	NC_000072.7
*Bmal1*	TGACCCTCATGGAAGGTTAGAA	GGACATTGCATTGCATGTTGG	NC_000073.7
*Clock*	CGGCGAGAACTTGGCATT	AGGAGTTGGGCTGTGATCA	NC_000071.7
*Cry1*	TGAGCTCGTGTCCGTTCG	CGGAGGACACGCATACCTTC	NC_000076.7

### 2.4 Immunofluorescent staining

After CSD, all experimental mice were anesthetized and perfused with cold 0.1 M PBS and 4% paraformaldehyde through the vascular system, and their brains were removed subsequently. After dehydration, the tissues were coated by Optimal Cutting Temperature Compound (Tissue‐Tek, 4583, SAKURA, Torrance, CA, USA) and sliced with the cryostat (CM‐1950S, Leica, Germany). A series of 10‐μm slices was cut coronally from the olfactory bulb to the cerebellum. We used coordination map to localize SCN (in the anterior hypothalamus, bregma −0.22 to −0.82 mm), RSC (the medial part of the dorsal cortex, bregma −1.34 to −2.46 mm), and LC (in the lower part of the 4th ventricle in the brainstem, bregma −5.34 to −5.80 mm) [34]. The tissues were incubated in the following antibodies overnight at 4°C: anti‐BMAL1 (ab3350, 1:1000, Abcam, Cambridge, UK), anti‐Cryptochrome I/CRY1 (ab104736, 1:200, Abcam, Cambridge, UK), anti‐KAT13D/CLOCK (ab3517,1:500, Abcam, Cambridge, UK), phospho‐Tau (Thr231) monoclonal antibody (AT180) (MN1040, 1:100, Thermo Fisher Scientific, Waltham, MA, USA), purified anti‐β‐Amyloid, 1–16 antibody (803001, 1:1000, Biolegend, San Diego, CA, USA), and monoclonal anti‐TH (tyrosine hydroxylase) antibody produced in mouse clone TH‐2 (T1299, 1:2000, Sigma‐Aldrich, St. Louis, MO, USA). Then, after washing three times in PBS, the slides were incubated with the secondary antibody anti‐rabbit IgG (H + L), F(ab′)2 Fragment (Alexa Fluor® 594 Conjugate) (8889S, 1:1000, Cell Signaling, Chicago, IL, USA), anti‐Mouse IgG (H + L), F(ab′)2 Fragment (Alexa Fluor® 488 Conjugate) (4408S, 1:1000, Cell Signaling, Chicago, IL, USA), anti‐rabbit IgG (H + L), F(ab′)2 Fragment (Alexa Fluor® 488 Conjugate) (4412S, 1:1000, Cell Signaling, Chicago, IL, USA) and anti‐mouse IgG (H + L), F(ab′)2 Fragment (Alexa Fluor® 594 Conjugate) (8890S, 1:1000, Cell Signaling, Chicago, IL, USA), for 1 h. Ten slices per animal (three random fields per slice) with the same reference position were photographed. The results were quantified by Image J.

### 2.5 Aβ level measurements

The hippocampus and cortex were dissected rapidly on ice and sonicated in ice‐cold lysis buffer (containing 50 mM Tris pH 7.4, 150 mM sodium chloride, 1% triton X‐100, 1% sodium deoxycholate, 0.1% sodium dodecyl sulfonate, and phosphatase inhibitors, such as sodium orthovanadate, sodium fluoride, ethylene diamine tetraacetic acid, leupeptin, etc., P10013B, Beyotime Institute of Biotechnology, China). The lysate was centrifuged at 12,000 × *g* for 10 min at 4°C. The supernatant was collected to determine human Aβ42/40 levels in AD mice by Amyloid beta 42 Human ELISA Kit (KHB3441, Thermo Fisher Scientific, Waltham, MA, USA) and Amyloid beta 40 Human ELISA Kit (KHB3481 Thermo Fisher Scientific, Waltham, MA, USA). The mouse Aβ42/40 levels in WT mice were also measured by Amyloid beta 42 Mouse ELISA Kit (KMB3441, Thermo Fisher Scientific, Waltham, MA, USA) and Amyloid beta 40 Mouse ELISA Kit (KMB3481, Thermo Fisher Scientific, Waltham, MA, USA) according to the manufacturer's instructions.

### 2.6 Statistical analysis

Statistical significance was determined using two‐way analysis of variance (ANOVA) with Tukey's multiple comparisons test and Student's *t* test by GraphPad Prism 7 (GraphPad Software Inc., La Jolla, CA, USA). All statistical data were expressed as mean ± standard error of the mean values (SEM). The *n* values in each figure legend represent the number of animals in the statistical analysis; *p* < 0.05 is considered statistically significant.

## 3 RESULTS

### 3.1 CSD causes cognitive decline in AD and WT mice

**FIGURE 1:**
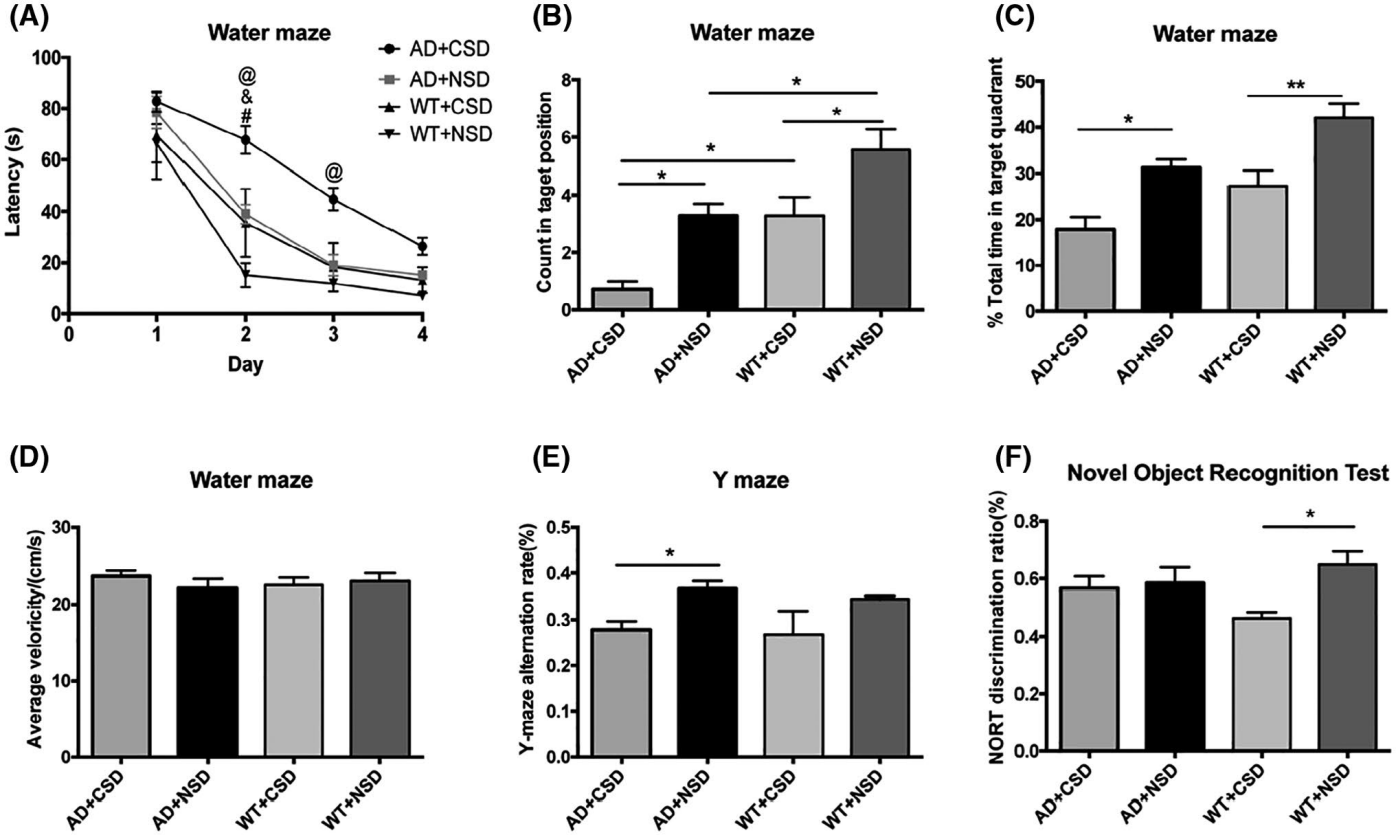
Learning and memory ability of 6–6.5 months old mice were impaired after CSD. After CSD, the escape latency of mice during the training period, *n* = 7 mice in each group (A). The number of times mice passed through the targeted position in quadrant after CSD, *n* = 7 mice in each group (B). The percentage of total time that the mice spent in the target quadrant during the Morris water maze task after CSD, *n* = 7 mice in each group (C). Average swimming speed of mice during Morris water maze task, *n* = 7 mice in each group (D). The Y maze alternation rate of AD and WT mice, *n* = 6 mice in AD + CSD group, *n* = 5 mice in AD + NSD group, *n* = 4 mice in WT + CSD mice, *n* = 8 mice in WT + NSD group (E). NORT discrimination ratio of AD and WT mice, *n* = 6 mice in AD + CSD and AD + NSD group, *n* = 5 mice in WT + CSD group, *n* = 10 mice in WT + NSD group (F). In (A) @: AD + CSD vs. AD + NSD, &: WT + CSD vs. WT + NSD, #: AD + NSD vs. WT + NSD. Data were the mean ± SEM values. *p < 0.05, **p < 0.01, ***p < 0.001, ****p < 0.0001, by two‐way ANOVA with Tukey's multiple comparisons test.

To evaluate the effect of CSD on the cognition of mice, we measured the cognitive behaviors of AD and WT mice. The results showed that the escape latency of AD + CSD mice was significantly longer than that of AD + NSD mice on day 2 (*p* < 0.0001) and day 3 (*p* < 0.001) during the training period, the escape latency of WT + CSD mice was significantly longer than that of WT + NSD mice on day 2 (*p* < 0.01), and the escape latency of WT + NSD mice was significantly shorter than that of AD + NSD mice on day 2 (*p* < 0.001). The number of times AD + CSD mice crossed the target position and the total time spent in the target quadrant during the Morris water maze task were significantly lower than that in AD + NSD mice (*p* < 0.05). The number of times WT + CSD mice crossed the target position (*p* < 0.05) and the total time spent in the target quadrant (*p* < 0.01) were also significantly lower than that of WT + NSD mice, whereas the number of times that WT + NSD mice crossed the target position was significantly higher than that of AD + NSD mice (*p* < 0.05). The number of times AD + CSD mice crossed the target position was significantly lower than that of WT + CSD mice (*p* < 0.05). No significant difference was found in the average swimming speed (Figure 1A–D). The Y‐maze alternation rate of AD + CSD mice was significantly lower than that of AD + NSD mice (*p* < 0.05) (Figure 1E), and the NORT discrimination ratio of WT + CSD mice was significantly lower than that of WT + NSD mice (*p* < 0.05) (Figure 1F). The two‐way ANOVA analysis showed an effect of genotype on the number of times the mice crossed the target position [*F*(1,24) = 19.82, *p* = 0.0002] and the total time spent in the target quadrant during the Morris water maze task [*F*(1,24) = 13.35, *p* = 0.0013]. These results suggested that CSD can cause cognitive decline but not motor impairment in both AD and WT mice.

### 3.2 CSD aggravates AD pathological changes

#### 3.2.1 Effect of CSD on Aβ pathology

**FIGURE 2:**
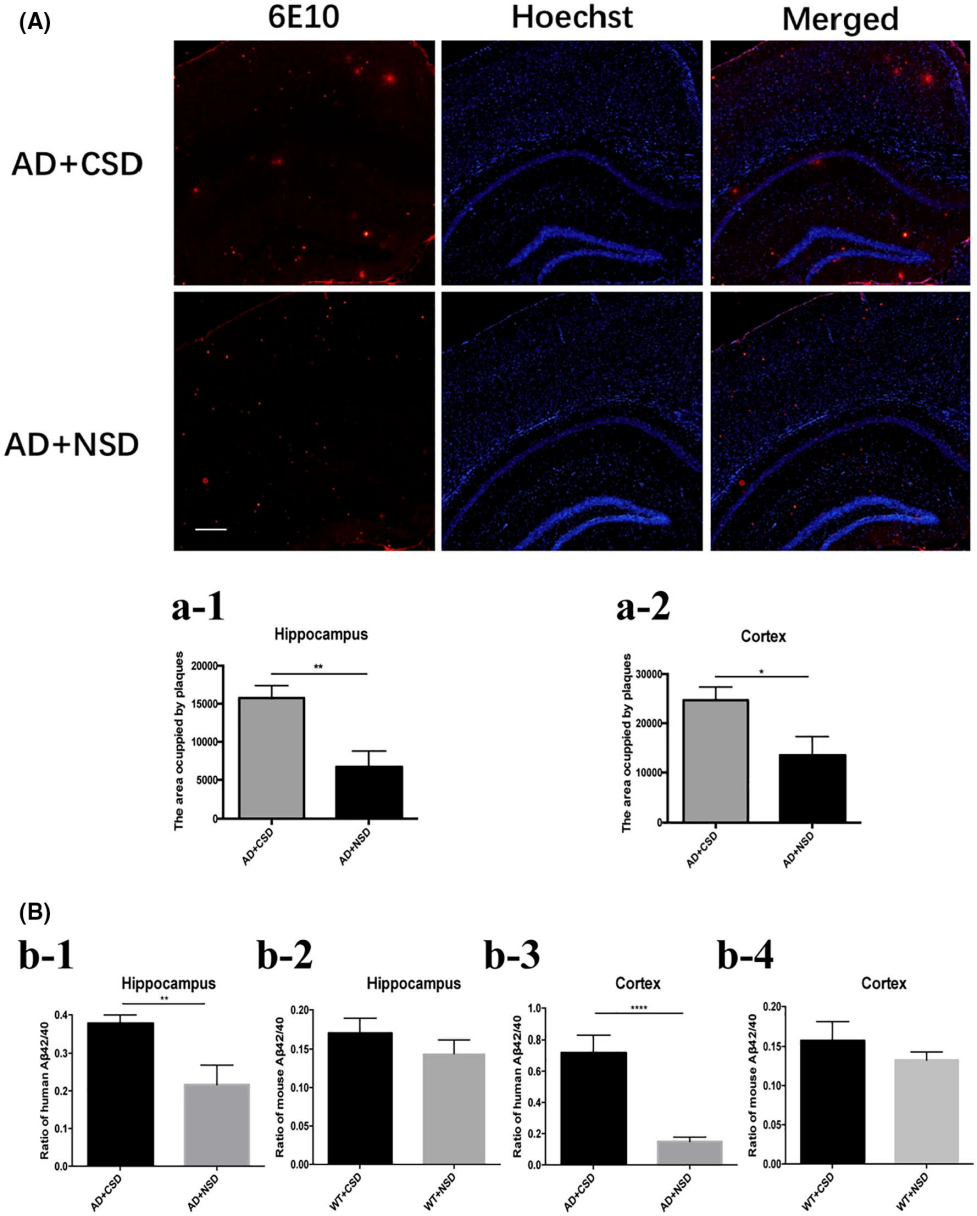
Deposition of Aβ plaque and the ratio of Aβ42/40 in hippocampus and cortex of 6–6.5 months old mice after CSD. The area occupied by plaques in mouse hippocampus and cortex after CSD, *n* = 6 mice in each group (A, a‐1, a‐2), in (a‐1): *t* = 3.421, df = 10, in (a‐2): *t* = 2.419, df = 10. The ratio of human Aβ42/40 and mouse Aβ42/40 in mice hippocampus and cortex, *n* = 4 mice in each group (B), in (b‐1): *t* = 4.936, df = 5, in (b‐2): *t* = 0.9654, df = 6, in (b‐3): *t* = 10.25, df = 6, in (b‐4): *t* = 1.027, df = 6. Scale bar: 100 μm. Data were the mean ± SEM values. *p < 0.05, **p < 0.01, ****p < 0.0001, by unpaired *t* test.

To confirm the effect of CSD on Aβ pathology, we measured the area occupied by plaques and the ratio of Aβ42/40 in mice hippocampus and cortex. The area occupied by plaques was increased significantly in the hippocampus (134%) (*p* < 0.01) and cortex (83%) (*p* < 0.05) of AD mice after CSD (Figure 2A, a‐1, a‐2). Plaque deposition was distributed throughout the cortex and hippocampus, but in the cortical region, plaques were more deposited in RSC. While the ratio of human Aβ42/40 was increased significantly in the hippocampus (*p* < 0.01) and cortex (*p* < 0.0001) of AD mice after CSD (Figure 2B), these results imply that CSD aggravated Aβ pathology in AD mice.

#### 3.2.2 Increased phospho‐Tau (p‐Tau) level after CSD was correlated with the change in BMAL1 expression

**FIGURE 3:**
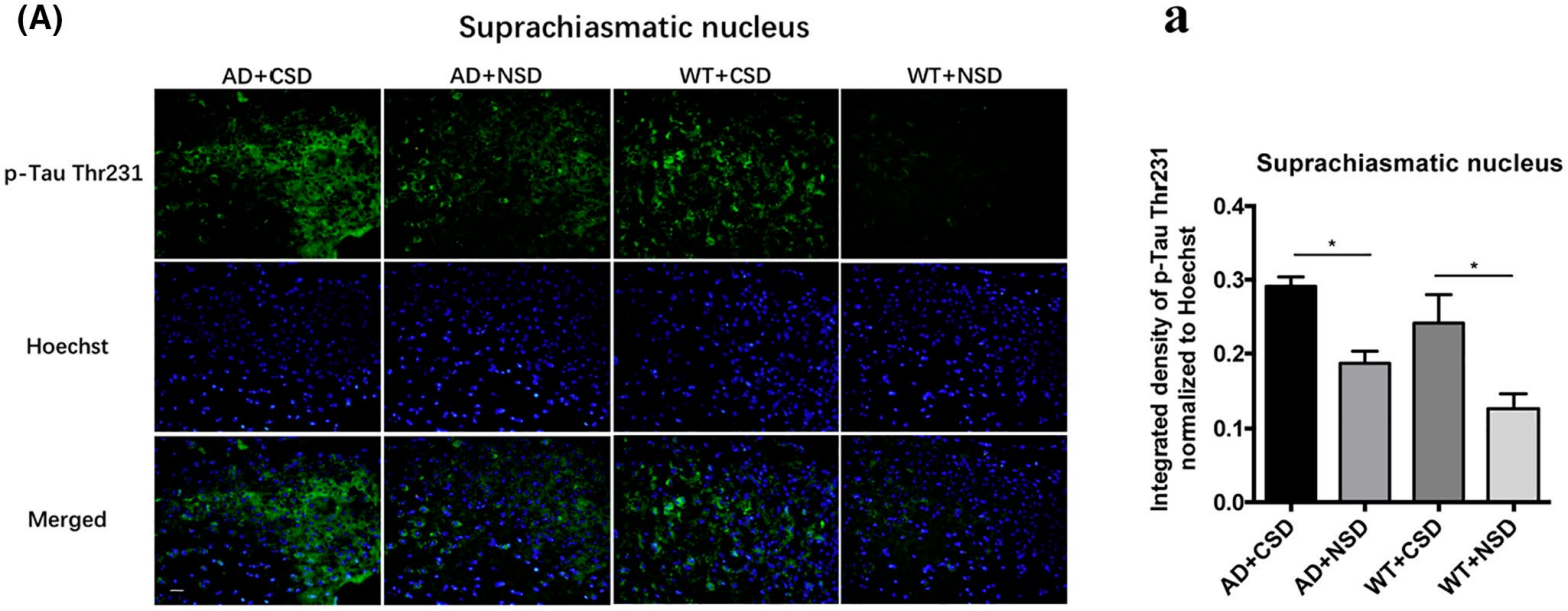
Immunofluorescent staining of p‐Tau Thr231 in SCN of 6–6.5 months old mice after CSD. The p‐Tau Thr231 staining in mouse SCN after CSD (A). Integrated density of p‐Tau Thr231 staining normalized to Hoechst staining was analyzed, *n* = 5 mice in each group (a). Scale bar: 20μm. Data were the mean ± SEM values. *p < 0.05, by two‐way ANOVA with Tukey's multiple comparisons test.

**FIGURE 4:**
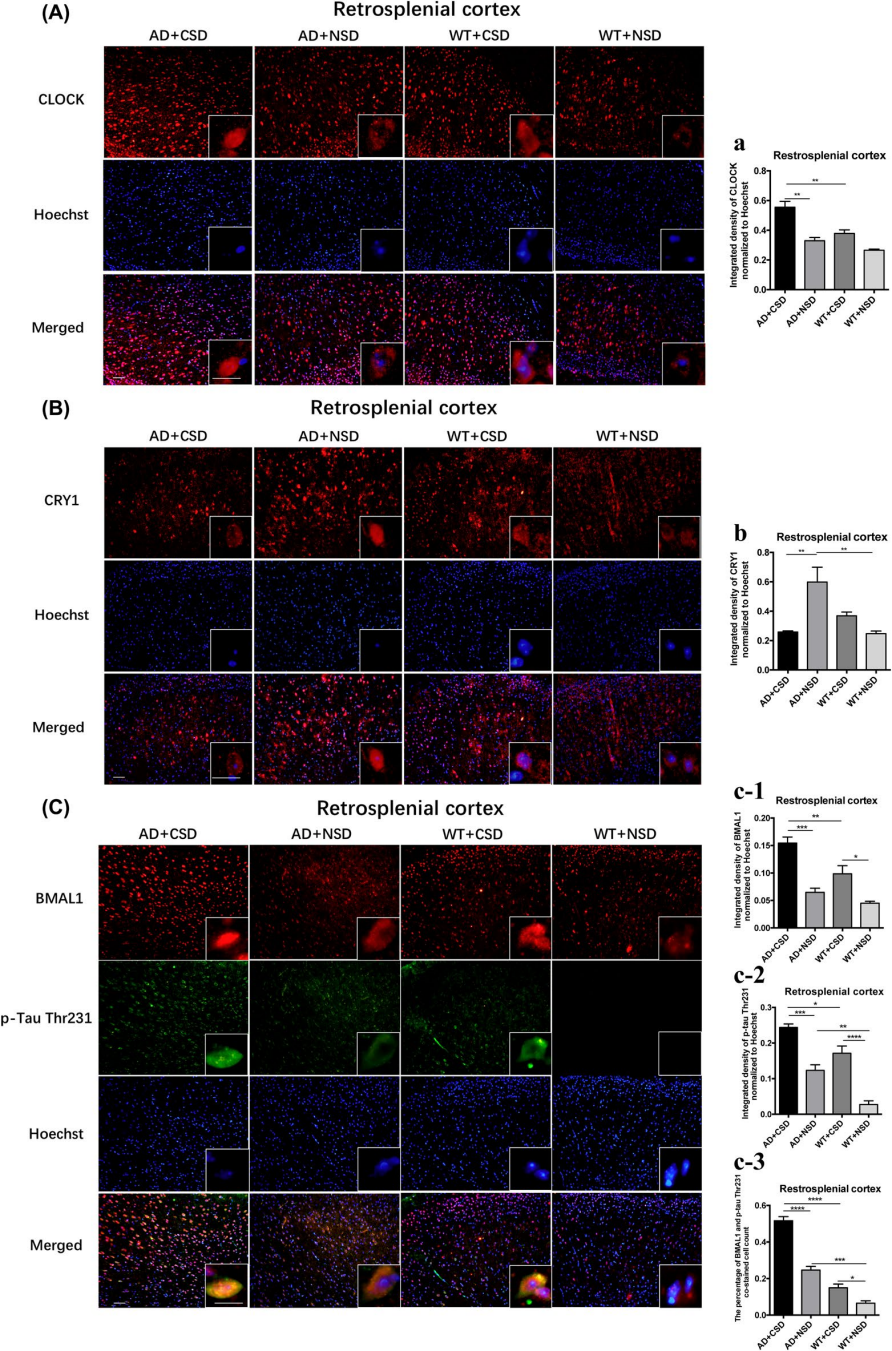
Immunofluorescent staining of CLOCK, CRY1, and double immunostaining of BMAL1 and p‐Tau Thr231 positive cells in RSC of 6–6.5 months old mice after CSD. The CLOCK staining in mouse RSC after CSD (A). Integrated density of CLOCK staining normalized to Hoechst staining was analyzed, *n* = 3 mice in each group (a). The CRY1 staining in mouse RSC after CSD (B). Integrated density of CRY1 staining normalized to Hoechst staining was analyzed, *n* = 4 mice in each group (b). Double immunostaining of BMAL1 and p‐Tau Thr231 in mouse RSC (C). Integrated density of BMAL1 staining normalized to Hoechst staining was analyzed, *n* = 4 mice in each group (c‐1). Integrated density of p‐Tau Thr231 staining normalized to Hoechst staining was analyzed, *n* = 4 mice in each group (c‐2). The percentage of BMAL1 and p‐Tau Thr231 co‐stained cells was analyzed, *n* = 4 mice in each group (c‐3). Left scale bar: 50μm, right scale bar: 20μm. Data were the mean ± SEM values. *p < 0.05, **p < 0.01, ***p < 0.001, ****p < 0.0001, by two‐way ANOVA with Tukey's multiple comparisons test.

Then, we analyzed the integrated density of p‐Tau Thr231 in the RSC, LC, and SCN after CSD. In SCN of AD (*p* < 0.05) and WT (*p* < 0.05) mice, significant p‐Tau accumulation was observed after CSD (Figure 3). The p‐Tau Thr231 level was increased significantly in the RSC of AD (*p* < 0.001) and WT (*p* < 0.0001) mice after CSD (Figures 4C, c‐2). The p‐Tau Thr231 level was increased significantly in the LC of AD (*p* < 0.01) mice (Figures 7A, a‐2). To investigate the potential connection between pathological changes of AD and clock genes, we performed double staining of p‐Tau Thr231 and BMAL1 in RSC after CSD. Fluorescent immunostaining combined with quantitative analysis clearly illustrated that the percentage of p‐Tau Thr231/BMAL1 co‐stained cells was increased significantly in the RSC of AD (*p* < 0.0001) and WT (*p* < 0.05) mice after CSD (Figures 4C, c‐3). These results suggested that CSD aggravated the pathological changes of AD in the circadian‐related nuclei and that there was a correlation between tau phosphorylation and altered BMAL1 expression.

### 3.3 Abnormal expression of clock genes in APP/PS1 mice in association with AD pathological changes

**FIGURE 5:**
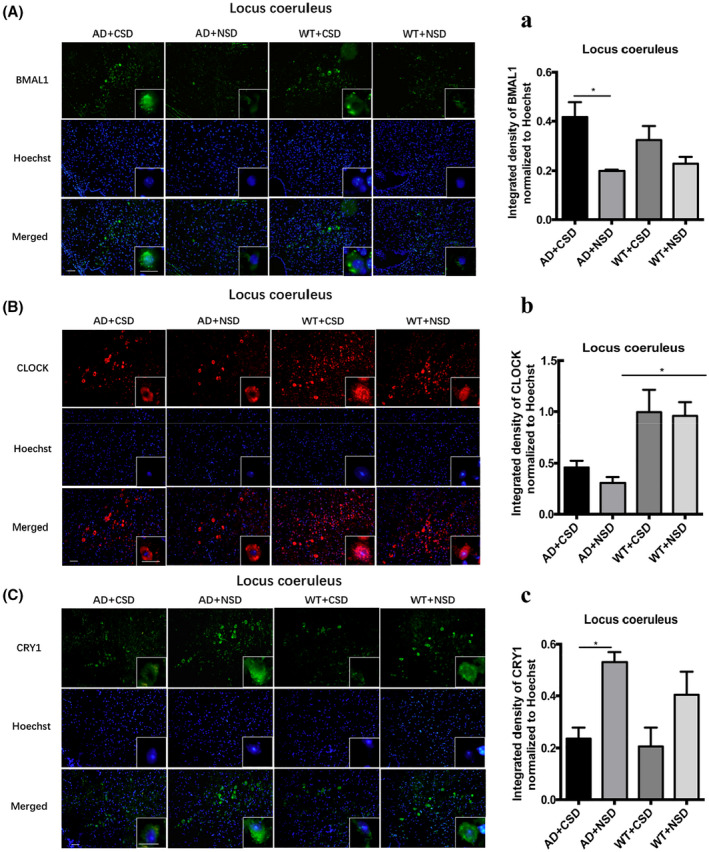
Immunofluorescent staining of BMAL1, CLOCK, and CRY1 in LC of 6–6.5 months old mice after CSD. The BMAL1 staining in mouse LC after CSD (A). Integrated density of BMAL1 staining normalized to Hoechst staining was analyzed, *n* = 4 mice in each group (a). The CLOCK staining in mouse LC after CSD (B). Integrated density of CLOCK staining normalized to Hoechst staining was analyzed, *n* = 4 mice in each group (b). The CRY1 staining in mouse LC after CSD (C). Integrated density of CRY1 staining normalized to Hoechst staining was analyzed, *n* = 4 mice in each group (c). Left scale bar: 50μm, right scale bar: 20μm. Data were the mean ± SEM values. *p < 0.05, by two‐way ANOVA with Tukey's multiple comparisons test.

**FIGURE 6:**
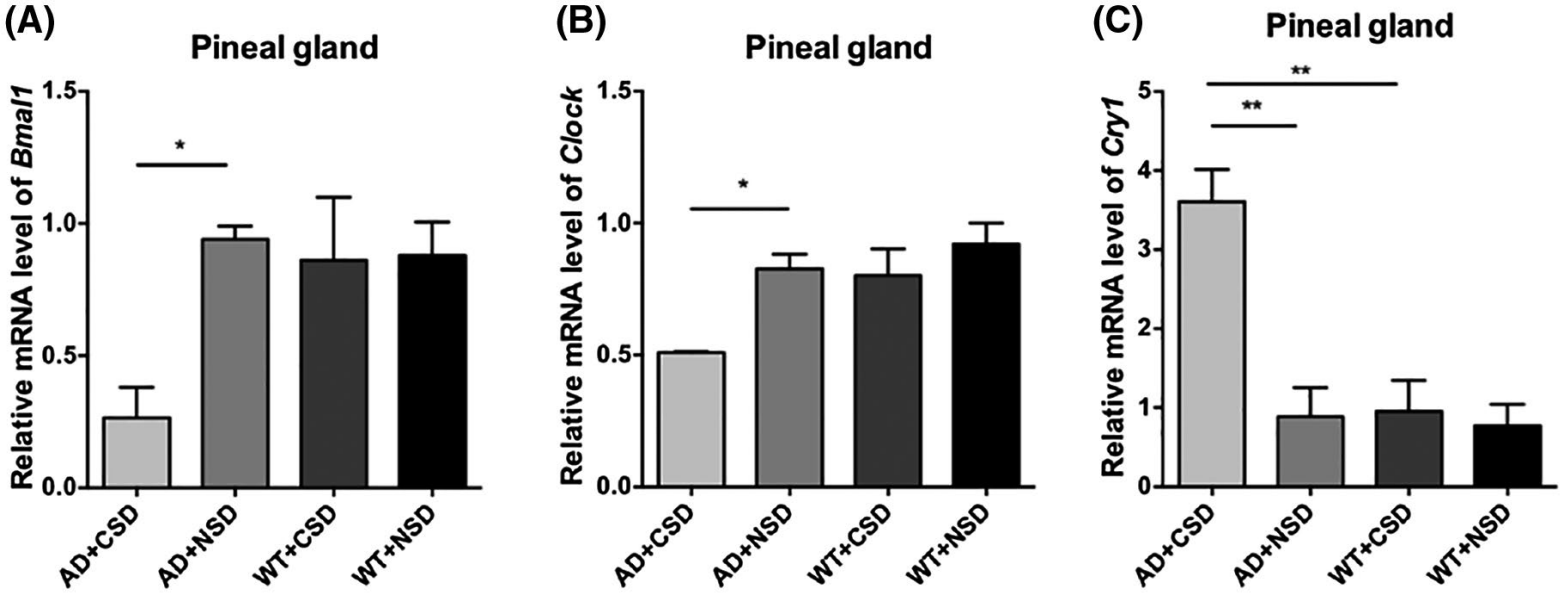
mRNA levels of clock genes were detected by real‐time PCR in pineal gland of 6–6.5 months old mice after CSD. The mRNA level of *Bmal1* in pineal gland of mice after CSD, *n* = 3 mice in each group (A). The mRNA level of *Clock* in pineal gland of mice after CSD, *n* = 3 mice in each group (B). The mRNA level of *Cry1* in pineal gland of mice after CSD, *n* = 3 mice in each group (C). Data were the mean ± SEM values. *p < 0.05, **p < 0.01, by two‐way ANOVA with Tukey's multiple comparisons test.

We found an abnormal expression of clock genes in the brain of AD mice aged 6–6.5 months. CRY1 level in the RSC (*p* < 0.01) of WT mice was significantly lower than that of the AD mice (Figures 4B, b). The percentage of the BMAL1 and p‐Tau Thr231 co‐stained cells in the RSC of AD + NSD mice was significantly higher than that of WT + NSD mice (*p* < 0.001) (Figures 4C, c‐3). Using a two‐way ANOVA, genotype demonstrated an influence on the expression of clock genes in the mouse brain, as in the level of BMAL1 in RSC [*F*(1,12) = 13.91, *p* = 0.0029] (Figures 4C, c‐1), level of CLOCK in RSC [*F*(1,8) = 21.57, *p* = 0.0017] and LC [*F*(1,12) = 19.26, *p* = 0.0009] (Figure 4A, a and 5B, b), the level of CRY1 in LC [*F*(1,12) = 5.180, *p* = 0.0420] (Figure 5C, c), and the mRNA levels of *Clock* [*F*(1,8) = 7.706, *p* = 0.0241] and *Cry1* [*F*(1,8) = 14.51, *p* = 0.0052] in the pineal gland (Figure 6B,C). These results demonstrated that the molecular clock was affected by APP/PS1 mutations. The abnormal BMAL1 expression in RSC was closely co‐localized with the expression of p‐Tau. It is suggested that the abnormal expression of BMAL1 may be related to tau phosphorylation.

### 3.4 Effect of CSD on the expression of clock genes in the RSC and LC

We measured the protein levels of BMAL1, CLOCK, and CRY1 in RSC and LC, respectively. The CRY1 level in RSC (*p* < 0.01) and LC (*p* < 0.05) of AD + CSD mice was significantly lower than that of AD + NSD mice (Figure 4B, b and 5C, c). BMAL1 level was significantly increased after CSD in RSC of both AD (*p* < 0.001) and WT (*p* < 0.05) mice, and the CLOCK level was increased significantly in RSC of AD (*p* < 0.01) mice after CSD (Figures 4A, a, C, c‐1). In the LC of AD (*p* < 0.05) mice, the BMAL1 level was significantly increased after CSD (Figure 5A, a). These results imply that CSD may cause the abnormal expression of clock genes in the brain of AD and WT mice.

### 3.5 Effect of CSD on the transcriptional levels of clock genes in the pineal gland

We measured the mRNA levels of *Bmal1*, *Clock*, *Cry1* in the pineal gland of mice. The results showed that mRNA levels of *Bmal1* and *Clock* decreased significantly after CSD (*p* < 0.05), but mRNA level of *Cry1* increased significantly in the pineal gland of the AD mice after CSD (*p* < 0.01). The mRNA level of *Cry1* in the pineal gland of AD + CSD mice was significantly higher than that of WT + CSD mice (*p* < 0.01) (Figure 6), indicating that the molecular clock in the pineal gland of AD mice is more susceptible to CSD than that of WT mice.

### 3.6 Effect of CSD on tyrosine hydroxylase (TH) level in LC

**FIGURE 7:**
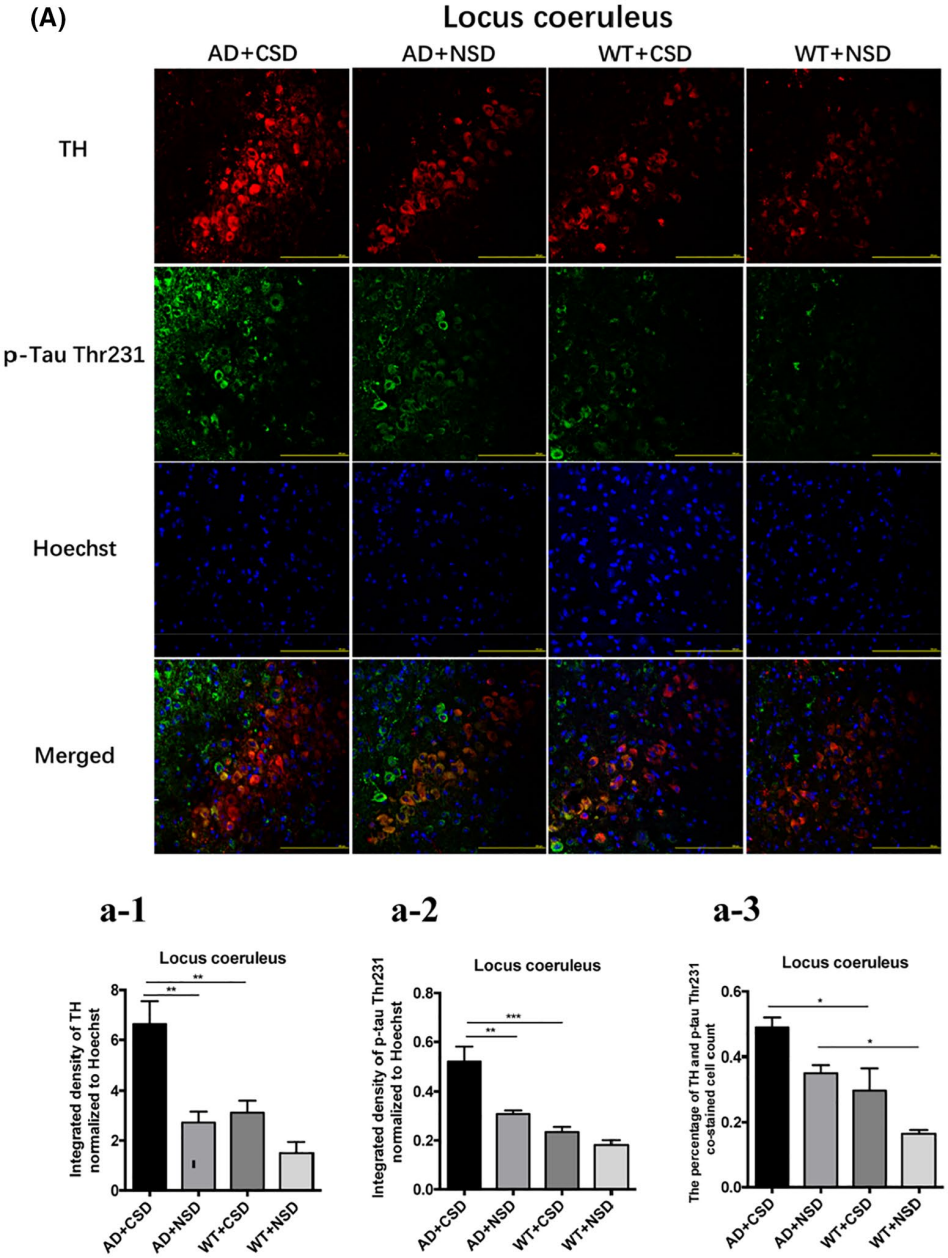
Double immunostaining of TH and p‐Tau Thr231 in LC of 6–6.5 months old mice after CSD. The double immunostaining of TH and p‐Tau Thr231 in mouse LC (A). Integrated density of TH staining normalized to Hoechst staining was analyzed, *n* = 4 mice in each group (a‐1). Integrated density of p‐Tau Thr231 staining normalized to Hoechst staining was analyzed, *n* = 4 mice in each group (a‐2). The percentage of TH and p‐Tau Thr231 co‐stained cells in mouse LC after CSD, *n* = 4 mice in each group (a‐3). Scale bar: 200μm. Data were the mean ± SEM values. *p < 0.05, **p < 0.01, ***p < 0.001, by two‐way ANOVA with Tukey's multiple comparisons test.

The TH expression cells of LC are arousal‐promoting cells. Therefore, we performed double immunostaining of TH and p‐Tau Thr231 in the LC of AD and WT mice after CSD. The results showed an increased level of TH in LC of AD mice after CSD (*p* < 0.01) (Figures 7A, a‐1). Using a two‐way ANOVA, CSD demonstrated an influence on the percentage of TH and p‐Tau Thr231 co‐stained cells [*F*(1,12) = 9.735, *p* = 0.0089] (Figures 7A, a‐3). The percentage of TH and p‐Tau Thr231 co‐stained cells in the LC of AD + NSD mice was significantly higher than that of WT + NSD mice (*p* < 0.05) (Figures 7A, a‐3). These results indicated that CSD can cause strong stimuli for the noradrenergic neurons. The accumulation of p‐Tau was most concentrated in LC, suggesting that the CSD‐induced tauopathy may mostly affect LC.

## 4 DISCUSSION

We reported that sleep changes may serve as an early sign of the pre‐pathological stage of AD [35]. CSD can lead to cognitive impairment in experimental mice and aggravate the pathological changes of AD [31], indicating that circadian disruption may be crucial in AD progression. It is a vacancy in the existing researches on whether the disruption of normal feedback loop of the molecular clock system is related to the pathological changes of AD after CSD. Nowadays, research on the causes of memory loss in AD is still focused on hippocampal dysfunction [36]. While the posterior cingulate region, which includes the RSC, has a direct connection with the hippocampus [37], and hippocampal ripples interact with RSC that plays a critical role in the acquisition and likely consolidation of new memories during sleep [38]. RSC also participates in spatial memory in conjunction with the anterior thalamic nucleus [22]. As reported, the MMP method of sleep deprivation is efficient to produce total REM suppression [32]. REM sleep may support the synaptic consolidation of memories in the cortex [39]. Therefore, CSD may affect the consolidation of memories in the cortex during sleep in the current study. We found that CSD affected the expression of clock genes in mouse RSC, and the abnormal BMAL1 expression in RSC was closely co‐localized with the expression of p‐Tau, suggesting that the abnormal expression of BMAL1 may be related to the abnormal phosphorylation of tau. It has been reported that increased wakefulness and sleep deprivation can increase ISF and CSF tau, tau spreading, and increase tau aggregation over longer periods of time [40]. Tau phosphorylation follows a circadian rhythm, when the circadian oscillation in body temperature was of smaller amplitude (when to sleep deprivation), the circadian changes in tau phosphorylation were abolished [41]. These studies have initially suggested that tauopathy follows the rhythm of the circadian clock, and our findings also provide evidence for this hypothesis. As reported, Aβ uptake by microglia varies with time of day in parallel with *Bmal1* expression, and pharmacologic treatment upregulated *Bmal1* mRNA level and increased fAβ1–42 uptake by BV‐2 cells relative to vehicle treatment in a dose‐dependent manner [42]. A possible explanation for the increase in BMAL1 level in RSC is a compensatory effect of increased Aβ deposition. There is a possibility that Aβ pathology is one of the main factors affecting the molecular clock.

SCN is a major pacemaker of the circadian system, driving circadian rhythmicity in other brain areas and peripheral tissues by sending them neural and humoral signals (such as melatonin), and most peripheral tissues and organs contain circadian oscillators [43]. However, under some circumstances (e.g., restricted feeding, jet lag, and shift work), they can desynchronize from SCN [44]. A study reported that the circadian noradrenergic regulation of the pineal by SCN is affected in both subjects with preclinical AD and patients with clinical AD [45]. The hCry1 mRNA is increased in the pineal glands of patients with AD, which is consistent with our findings in CSD‐treated mice. These changes may be caused by a diminished output of SCN from the early AD stages onward [46]. Tauopathy in SCN may disrupt normal circadian clock function at both behavioral and molecular levels [47]. Interestingly, in this study, we found that CSD can lead to an increase in p‐Tau level in the SCN, which suggests that the accumulation of p‐Tau in SCN may be leading to the disruption of circadian clock function after CSD treatment. The pathological changes of SCN are one of the possible factors leading to the abnormal expression of clock genes in non‐SCN (Figure S2). Expression levels of different clock genes may have different rhythms in different tissues [43, 48]. The changes in the expression of clock genes in the pineal gland after CSD are different from those in other nuclei, which may be related to the different roles of these nuclei in regulating circadian rhythm.

The noradrenergic neurons of the LC projection system provide the primary source of NE for the forebrain, mediating memory and attention [19, 49]. The neurons in the dorsal division of the LC also contribute to the regulation of the forebrain clock, at least in part, through the targeted release of NE into the cortical area [50]. In the LC of AD mice, we found a significant increase in BMAL1 and p‐Tau levels after CSD. The TH level increased significantly in the LC of AD and WT mice after CSD, which is consistent with the finding of a previous study on the expression level of TH after sleep deprivation. Under chronic stress, the adrenergic nervous system was markedly activated, as the expression of TH was enhanced [51]. It is believed that sleep deprivation provided stronger stimuli for the noradrenergic neurons, which may increase the release of NE from the terminals and led to a decrease in NE concentrations and finally led to the increased level of TH [52, 53]. As reported, endogenous Aβ42 is localized to TH immunoreactive somatodendritic profiles of the LC and dopamine‐β‐hydroxylase immunoreactive axon terminals of the infralimbic medial prefrontal cortex, and the reductions in NE correspond to reductions in endogenous Aβ42 [54]. The increase of TH level after CSD may be related to the aggravation of Aβ pathology. Other researchers suggested that hyperactive noradrenergic signaling in AD as a critical element linking Aβ to the pathogenic GSK3β/tau cascade [55]. TH mRNA and protein levels in mice peak at night and dip during the day, similarly to *Bmal1* [56]. In the current study, the stress caused by CSD is an important factor leading to the increase of TH level. At the same time, the simultaneous increase of TH level and BMAL1 level after CSD provided evidence that there might be a connection between the TH level and the expression of clock genes. Other researchers have also found that several key DA‐regulating genes are proposed to be controlled by clock genes, such as *Clock* and *Rev*‐*erbɑ* [56, 57]. NE has a specific role in the regulation of REM sleep [58, 59]. The increase in NE level after sleep deprivation may further cause REM sleep loss, increase Na‐K ATPase activity, and finally lead to the alteration of brain excitability [60]. LC can regulate the forebrain activity state, the enhanced LC discharge contributes to stress‐induced activation of forebrain EEG and the increase of arousal [19]. Under conditions of higher arousal (e.g., stress), associated with increased rates of NE release, stimulation of ɑ1‐receptors impairs working memory [19]. LC is one of the first brain structures to accumulate p‐Tau inclusions in AD [61, 62, 63], and LC can seed tau formation and its transneuronal propagation to most areas of the greater cortex via major afferents and efferent of the LC [64]. Therefore, understanding how tauopathy develops in LC may help us to better define the pathogenesis and development of AD.

Clock genes (such as *Bmal1*) are directly involved in the cellular antioxidant responses [65]. The disruption of *Bmal1* impairs blood–brain barrier integrity via pericyte dysfunction [66]. Clock gene deletion has been found to result in pronounced astrocyte activation, indicating that clock genes can also directly regulate neuroinflammation [67]. At the same time, local *Bmal1* deletion in the brain induces ApoE expression [68]. The alteration of BMAL1 methylation in AD and Aβ‐induced degradation of BMAL1 and CBP has been considered a possible cause of circadian rhythm disorder [69, 70]. Several studies have shown that single nucleotide polymorphisms in CLOCK and BMAL1 are associated with an increased risk of AD or PD [71, 72, 73]. After CSD, we observed abnormal expression of clock genes in the LC, RSC, and pineal gland of AD mice, suggesting the occurrence of abnormal molecular clock oscillation. Abnormal molecular clock oscillation may influence disease progression through its regulatory effects on inflammation, oxidative stress, blood–brain barrier integrity, and other functions, which collectively may promote disease progression through its influence on the pathology of Aβ and tau. These changes were not significant in WT mice compared with AD mice, suggesting that APP/PS1 mutations have an effect on the molecular clock, which may be because of Aβ pathology.

In summary, we observed abnormal expression of clock genes in the brain of mice after CSD, which might provide important information for the study of the molecular mechanism of the circadian rhythm disorder caused by genetic and environmental factors. Future research may focus on whether the effect of CSD on the expression of clock genes is because of the phase shift or the loss of the circadian rhythm. There may be a potential link between the molecular clock, Aβ pathology, tauopathy, and the noradrenergic system. The abnormal expression of clock genes because of APP/PS1 mutations may be the molecular basis of the disruption of the circadian rhythm in AD patients with a genetic predisposition. The results of this study provided new insights into the potential link between the disruption of circadian rhythm and the development of AD.

## CONFLICT OF INTEREST

The authors declare no conflicts of interest.

## AUTHOR CONTRIBUTIONS

Weidong Le contributed to research concept and support; Long Niu, Feng Zhang, Xiaojiao Xu, and Yuting Yang contributed to experiments and data analysis; Song Li and Hui Liu provided technical support; Long Niu and Feng Zhang contributed the initial writing of the manuscript; and Weidong Le contributed the revision of the manuscript.

## Supporting information

Fig S1‐S2
**FIGURE S1** The sleep deprivation was performed using a modified multiple platform method. CSD‐treated mice were placed in a water tank with small platforms (with a diameter of 3 cm and height of 5 cm) (A). NSD‐treated mice were placed in a water tank with a platform diameter of 11.5 cm, and the other conditions were similar to those of CSD groups (B)
**FIGURE S2** Abnormal expression of clock genes in sleep‐related nuclei after CSD and its potential effect on the regulation of circadian rhythm. After CSD, the abnormal expression of clock genes in AD mice pineal gland, LC and RSC may be related to the dysfunction of SCN

## Data Availability

The data that support the findings of this study are available from the corresponding author upon reasonable request.
