# Supplementary material for: Chronic sleep deprivation altered the expression of circadian clock genes and aggravated Alzheimer's disease neuropathology
Source: Brain Pathol. 2021 Oct 20;32(3):e13028. doi: 10.1111/bpa.13028 (PMC9048513; doi:10.1111/bpa.13028)
Supplement: Supplementary file 1 — Fig S1‐S2 FIGURE S1 The sleep deprivation was performed using a modified multiple platform method. CSD‐treated mice were placed in a water tank with small platforms (with a diameter of 3 cm and height of 5 cm) (A). NSD‐treated mice were placed in a water tank with a platform diameter of 11.5 cm, and the other conditions were similar to those of CSD groups (B) FIGURE S2 Abnormal expression of clock genes in sleep‐related nuclei after CSD and its potential effect on the regulation of circadian rhythm. After CSD, the abnormal expression of clock genes in AD mice pineal gland, LC and RSC may be related to the dysfunction of SCN [file BPA-32-e13028-s001.docx]

**SUPPLEMENTARY INFORMATION**


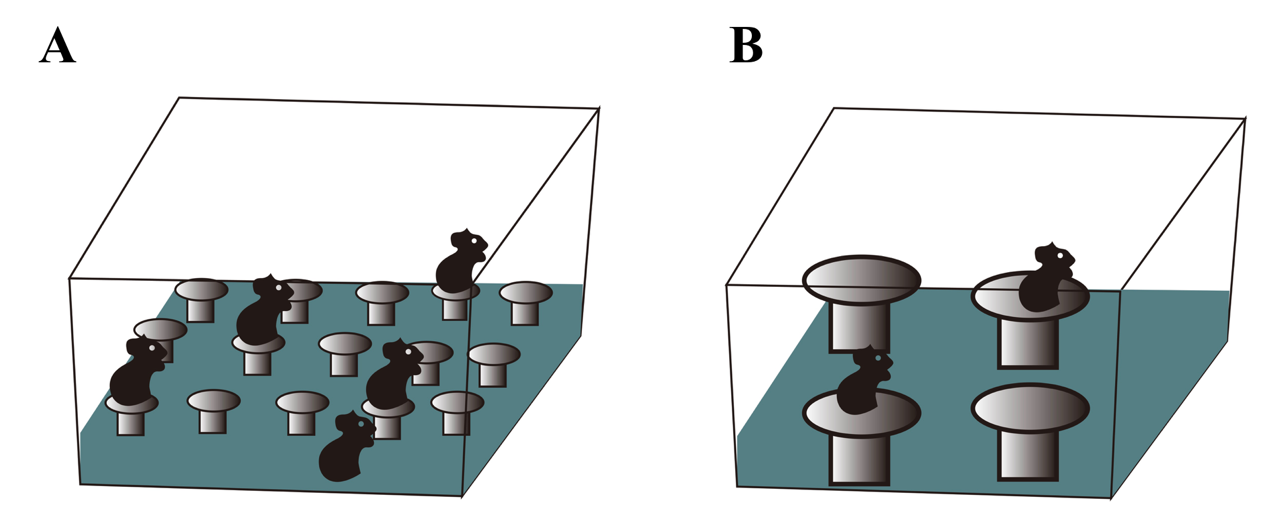
**Supplemental Figure 1.** The sleep deprivation was performed using a modified multiple platform method. CSD-treated mice were placed in a water tank with small platforms (with a diameter of 3 cm and height of 5 cm) **(A)**. NSD-treated mice were placed in a water tank with a platform diameter of 11.5 cm, and the other conditions were similar to those of CSD groups **(B)**.


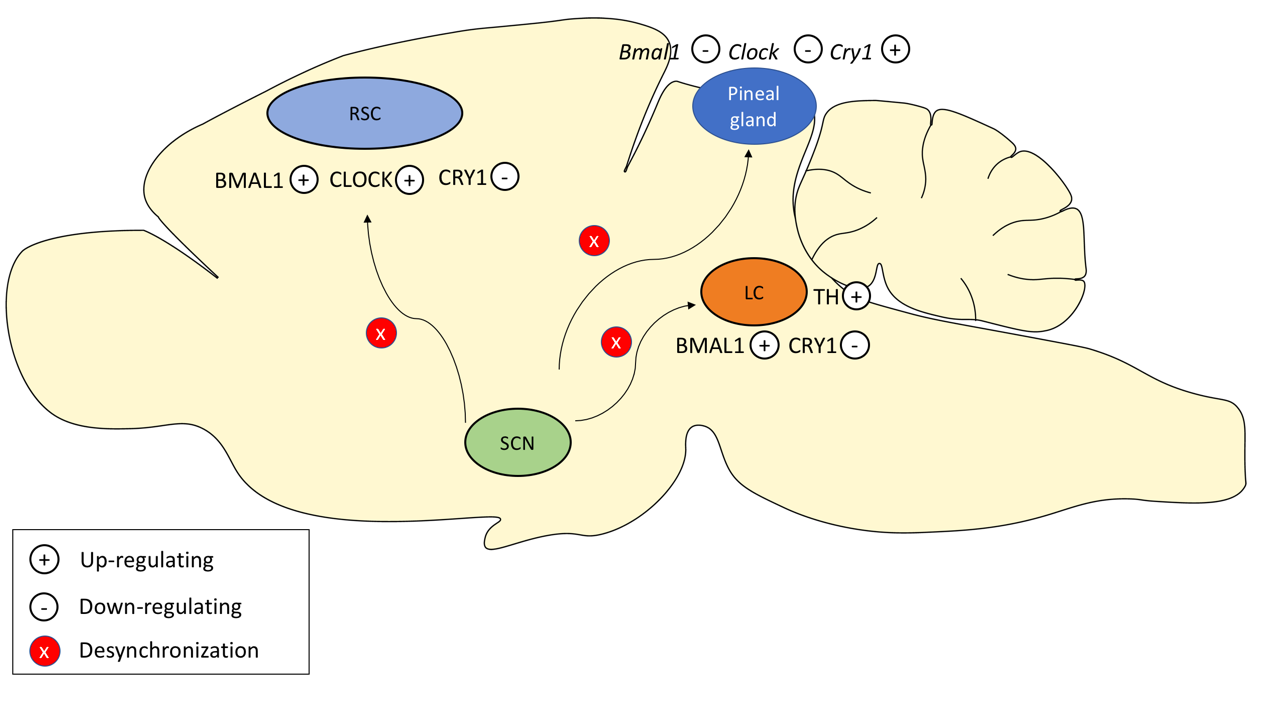


**Supplemental Figure 2.** Abnormal expression of clock genes in sleep-related nuclei after CSD and its potential effect on the regulation of circadian rhythm. After CSD, the abnormal expression of clock genes in AD mice pineal gland, LC and RSC may be related to the dysfunction of SCN.
